# Cordycepin Isolated from *Cordyceps militaris*: Its Newly Discovered Herbicidal Property and Potential Plant-Based Novel Alternative to Glyphosate

**DOI:** 10.3390/molecules24162901

**Published:** 2019-08-09

**Authors:** Tran Ngoc Quy, Tran Dang Xuan, Yusuf Andriana, Hoang-Dung Tran, Tran Dang Khanh, Rolf Teschke

**Affiliations:** 1 Graduate School for International Development and Cooperation, Hiroshima University, Hiroshima 739-8529, Japan; 2 Can Tho University, Can Tho City 902070, Vietnam; 3 Faculty of Biotechnology, Nguyen Tat Thanh University, 298A-300A Nguyen Tat Thanh Street, Ward 13, District 4, Ho Chi Minh 72820, Vietnam; 4 Agricultural Genetics Institute, Pham Van Dong Street, Hanoi 122000, Vietnam; 5 Center for Expert, Vietnam National University of Agriculture, Hanoi 131000, Vietnam; 6 Department of Internal Medicine II, Division of Gastroenterology and Hepatology, Klinikum Hanau, 63450 Hanau, Germany; 7 Academic Teaching Hospital of the Medical Faculty, Goethe University Frankfurt/ Main, D-60590 Frankfurt/Main, Germany

**Keywords:** *Cordyceps militaris*, allelopathy, allelochemical, cordycepin, glyphosate, inhibition, germination, growth, *Raphanus sativus*, plant growth inhibitor, column chromatography

## Abstract

There is currently much interest in finding new phytochemicals among plants and fungi as nature-based alternatives to replace problematic herbicides such as glyphosate, which are preferentially used in agricultural production. We discovered striking herbicidal potency in *Cordyceps militaris* (L.) and identified cordycepin as its principal plant growth inhibitor. Cordycepin obtained as an ethyl acetate extract was subjected to column chromatography and evaluated for its bioassay-guided phytotoxic capacity against *Raphanus sativus* (radish), showing a maximum inhibition on germination and growth of radish (IC_50_ = 0.052–0.078 mg/mL). Gas chromatography-mass spectrometry (GC-MS) (*m*/*z*: 251.2) and liquid chromatography-electrospray ionization-mass spectrometry (LC-ESI-MS) ([M + Na]^+^ *m*/*z*: 274.1; [M + H]^+^ *m*/*z*: 252.1) analyses confirmed cordycepin as the major component of the tested column fraction (55.38%). At 0.04 mg/mL, cordycepin showed 3.8–5.9- and 3.3–3.7-fold greater inhibition of the germination and growth of radish than benzoic acid (BA) and glyphosate, respectively. Compared with BA, isolated cordycepin reduced plant chlorophyll and carotenoid contents (2.0–9.5 -fold), while proline, total phenolic and total flavonoid contents were increased 1.2–1.8-fold. Finally, cordycepin promoted electrolyte leakage and malondialdehyde accumulation in radish aerial parts. Thus, cordycepin successfully isolated from *Cordyceps militaris* is a highly potent plant growth inhibitor with pending worldwide patent and may become a potential plant-based novel alternative to the disputed glyphosate.

## 1. Introduction

In settings of agriculture, botany, politics, and medicine, much controversy has emerged on the overuse and risks of herbicides in agricultural production, and discussions have recently focused on the development of biological controls using an allelopathy approach. Allelopathy is a biological phenomenon by which one species produces phytochemicals that affect the growth and development of other nearby plant species [1]. These phytochemicals, also called allelochemicals, are produced principally as secondary metabolites of plants and microorganisms [2]. As natural toxins, allelochemicals affect a target plant by morphological, cytological, physiological, and biochemical changes in plant aerial parts and roots [3]. They inhibit cellular processes in target plants such as cell division, membrane permeability, stomatal closure, absorption of nutrients, photosynthesis, ATP synthesis, metabolic processes and gene expression [4].

Some secondary metabolites produced by fungi are also allelochemicals [5,6,7,8]. The phytotoxicity of fungi is dependent on the quantity, strength and variety of allelochemicals [9]. Utilization of phytochemicals from fungi for weed control in agriculture has received increasing attention [10]. Fungi containing phytotoxic compounds might be a promising natural tool to manage weeds and pathogenic infestation [9]. Therefore, isolation and identification of the active herbicidal substances from fungal metabolites warrant further efforts. *Cordyceps militaris* (L.) Link is a fungus belonging to the class Ascomycetes [11]. This fungus exhibits a wide spectrum of pharmacological activities, including anti-stress, antifatigue [12], antioxidant [13], antifungal, and anticancer [14]. The fruiting body of *C. militaris* contains various active components such as cordycepin, adenosine, polysaccharides, fatty acids, amino acids, and other chemicals [15]. Among of them, cordycepin, a nucleoside analog (3′-deoxyadenosine), exhibits the most biological and pharmacological functions such as antineoplastic, antioxidant, and anti-inflammatory activities, tumor cell apoptosis and a decrease in tumor cell proliferation [16,17,18].

To compare the phytotoxic power of isolated natural phytotoxins, benzoic acid (BA) is commonly used as a standard control [19]. BA is applied as a commercial herbicide [20] and is reported to modify in indicator plants physiological processes such as the net photosynthetic rate, stomatal conductance, nutrient uptake, and resulted in growth inhibition [21,22,23]. Recently, Andriana et al. [24] demonstrated that BA inhibited the germination of radish at a concentration of 1.0 mg/mL in bioassays.

Our present study evaluates the phytotoxic potential of *C. militaris* on *R. sativus* (radish) compared with BA in a search for nature-based alternatives for disputed herbicides, such as glyphosate. Results of isolation, application of different extraction methods to obtain a maximum yield of cordycepin, and bioassay-guided phytotoxicity evaluations have been provided. More specifically, the inhibition of cordycepin on physiological and biochemical responses of radish seedlings including total phenolic contents (TPC), total flavonoid contents (TFC), chlorophyll and carotenoid contents, electrolyte leakage, lipid peroxidation, and proline contents were also investigated.

## 2. Results

### 2.1. Inhibitory Effects of Crude Extracts of C. militaris Fruiting body

The inhibitory levels of crude extracts obtained from various selected solvents are shown in Table 1. In general, all extracts inhibit germination and growth of radish, however, the ethyl acetate (EtOAc) extract presents the highest inhibition of germination and elongation of root and shoot of radish (IC_50_ values = 0.235, 0.127 and 0.096 mg/mL, respectively). The inhibition of *C. militaris* extracts followed the order EtOAc > aqueous residue > chloroform > hexane, suggesting that the EtOAc extract contains more potent allelochemicals than other extracts. The EtOAc extract was thus selected for further fractionation.

### 2.2. Effects of Fractions from the EtOAc Extract of C. militaris Fruiting Body

**Figure 1:**
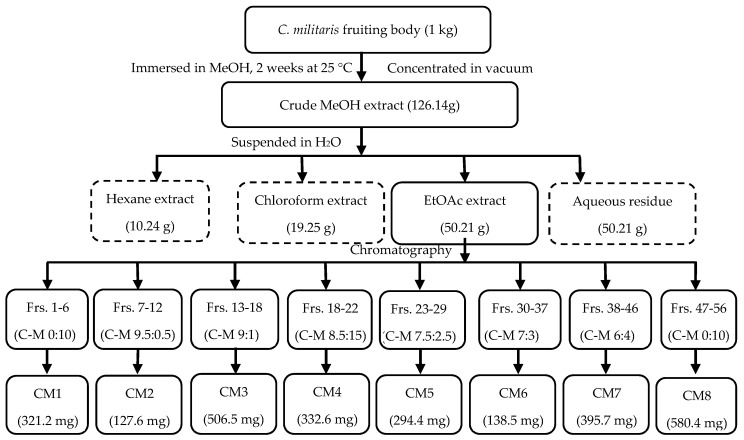
Procedure of extraction and fractionation of *C. militaris* fruiting-bodies (Frs: fraction, C: chloroform, M: methanol).

The separation of fractions from *C. militaris* was conducted following the procedure illustrated in Figure 1. Finally, the EtOAc extract was separated by column chromatography (CC) to get eight fractions with their corresponding yields (CM1 to CM8).

The phytotoxic effects of the isolated fractions, which were evaluated on the germination and elongation of roots and shoots of radish, are shown in Table 2. Eight fractions obtained from the EtOAc extract of *C. militaris* showed various levels of inhibitions. The CM4 fraction presents the strongest inhibitory levels in germination, root elongation and shoot height (IC_50_ = 0.078, 0.053 and 0.052 mg/mL, respectively). Generally, the greater effect of CM4 indicates that this fraction preferentially contains strong plant growth inhibitors, more so than the other fractions.

The inhibitory effects of the CM4 fraction were compared with BA and synthetic cordycepin (Table 3). CM4 and cordycepin both exert strong suppression of the germination of radish from 4.6- to 5.9-fold). Similarly, CM4 and cordycepin also showed much greater inhibition on the elongation of roots (3.5- to 4.5-fold) and shoots (3.5- to 3.8-fold) than BA. In general, it was concluded that the inhibitory capacities of CM4 and cordycepin were both greater than that of BA, by 3.5- to 5.9-fold on the germination and emergence of radish. The inhibitory effect of synthetic cordycepin was found to be greater than that of CM4, but the difference was not statistically different (Table 3). Besides, compared to the previous study [25], cordycepin was stronger than glyphosate by 3.3- to 3.7-fold on the germination and growth of radish (Table 3).

### 2.3. Physiological Responses to the CM4 fraction, Cordycepin and Benzoic Acid

#### 2.3.1. Chlorophyll and Carotenoid Contents

**Figure 2:**
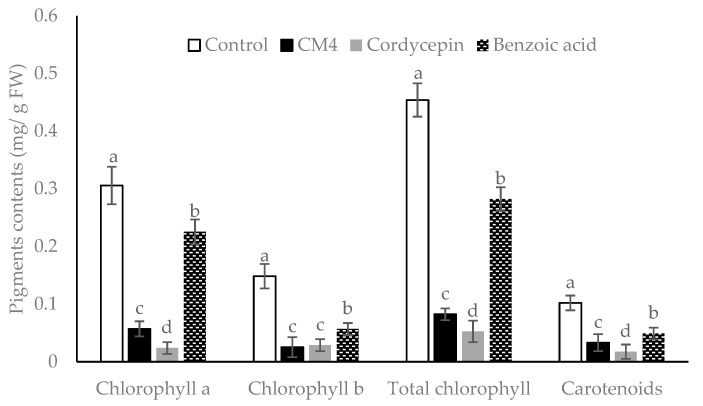
Pigment contents of radish treated with CM4 fraction, synthetic cordycepin and BA at 0.04 mg/mL. Columns with different superscript letters (^a,b,c,d^) in bars showed significant differences at *p* < 0.05 by Fisher’s test.

The CM4 fraction, synthetic cordycepin, and BA reduced the chlorophyll and carotenoid accumulations of radish at 0.04 mg/mL, although the inhibitory levels varied (Figure 2). The CM4 fraction significantly decreases chlorophylls (a, b, and total chlorophylls) and carotenoid contents by 80.65%, 80%, 80.43%, and 70%, respectively, as compared to the control (Figure 2). Cordycepin reduces quantities of chlorophylls and carotenoids significantly more than both CM4 and BA. Generally, the reduction of accumulation levels is as follows: cordycepin > CM4 > BA. Findings from Table 3 and Figure 2 indicate that cordycepin acts as an allelochemical, which strongly reduces germination and growth of radish, as well as the accumulations of chlorophylls a and b and carotenoids. Both CM4 and cordycepin caused significantly stronger inhibition than BA. The CM4 fraction contained the potent plant growth inhibitors and thus was further analyzed by GC-MS and LC-ESI-MS.

#### 2.3.2. Electrolyte Leakage

**Figure 3:**
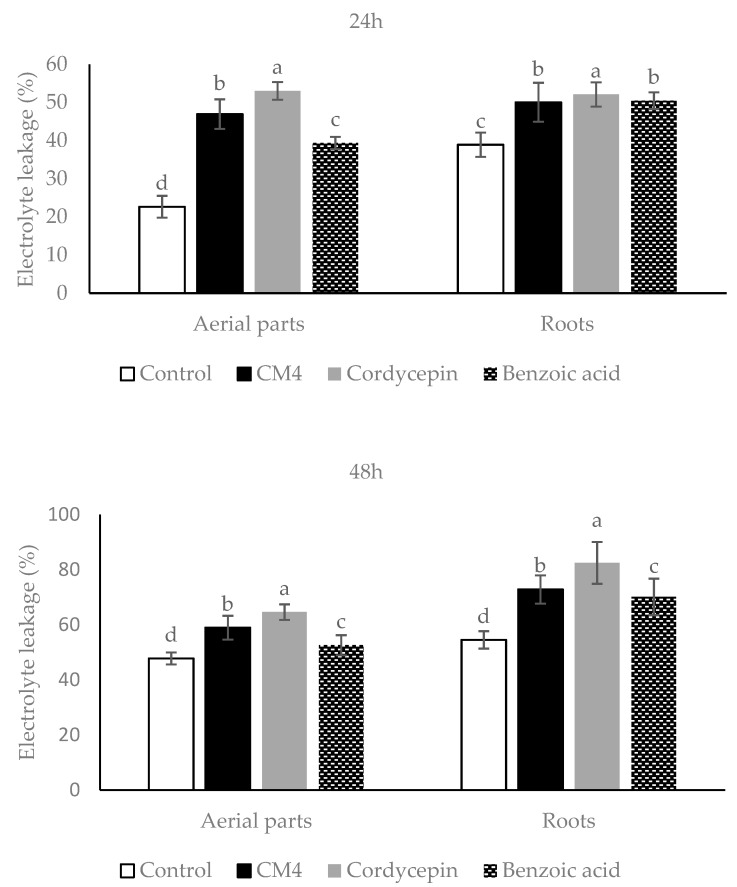
Electrolyte leakage (%) of radish treated by CM4 fraction, synthetic cordycepin and BA at 0.04 mg/mL. Columns with similar superscript letters (^a,b,c,d^) in bars were not significantly different according to Fisher’s test (*p* <0.05).

Compared to controls, the levels of electrolyte leakage (EL) of the roots and aerial parts of radish caused by CM4, synthetic cordycepin and BA are variable (Figure 3); with all compounds and after 24 and 48 h, EL values are significantly higher compared to controls. In addition, the EL value provoked by synthetic cordycepin was significantly greater than the one caused by CM4 and BA, while CM4 caused markedly higher EL than BA (Figure 3). The conclusion is reached that treatment of synthetic cordycepin, BA, and CM4 promoted EL values of radish, substantiating their potential herbicidal activity.

#### 2.3.3. Lipid Peroxidation

**Figure 4:**
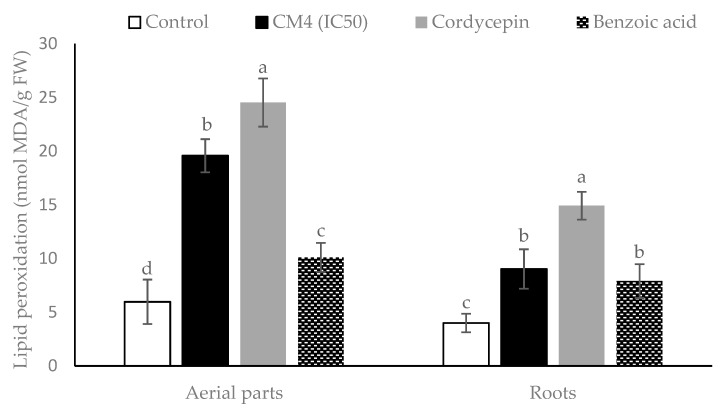
Lipid peroxidation expressed as malondialdehyde (MDA) accumulation in radish among control and treatments (CM4 fraction, synthetic cordycepin and BA) at 0.04 mg/mL. Columns with different superscript letters (^a,b,c,d^) in bars indicate significant differences at *p* < 0.05 by Fisher’s test.

The responses of *R. sativus* to the CM4 fraction, BA, and synthetic cordycepin in malondialdehyde (MDA) accumulation are illustrated in Figure 4. All treatments significantly promote MDA accumulation in both the aerial parts and roots. The CM4 fraction remarkably increases the MDA content of radish by 3.28- and 2.26-fold in the aerial parts and roots, compared to controls (Figure 4). Furthermore, lipid peroxidation accumulation in the radish by the CM4 fraction is higher than accumulation by BA in the aerial parts. However, synthetic cordycepin causes maximum level of lipid peroxidation as compared with either CM4 or BA (Figure 4).

### 2.4. Biochemical Responses to the CM4 Fraction, Cordycepin and Benzoic acid

#### 2.4.1. Total Phenolic Contents

**Figure 5:**
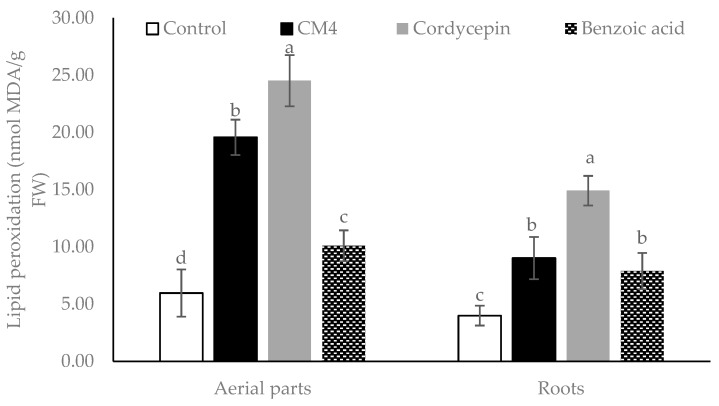
The changes of total phenolic accumulation in the roots and aerial parts of radish among control, CM4 fraction, synthetic cordycepin and BA at 0.04 mg/mL. Columns with similar superscript letters (^a,b,c,d^) were not significantly different at *p* < 0.05 by Fisher’s test.

The effects of CM4, BA, and synthetic cordycepin on the total phenolic contents (TPC) of radish are variable (Figure 5). CM4 treatment significantly enhanced the TPC in the roots and aerial parts of the radish by 87.69% and 42.95%, respectively, as compared with controls. The TPC by CM4 is similar to synthetic cordycepin in the aerial parts as they are markedly higher than by BA, however, the TPC caused by cordycepin is highest in the roots of the radish and significantly higher than that caused by both CM4 and BA (Figure 5).

#### 2.4.2. Total Flavonoid Contents

**Figure 6:**
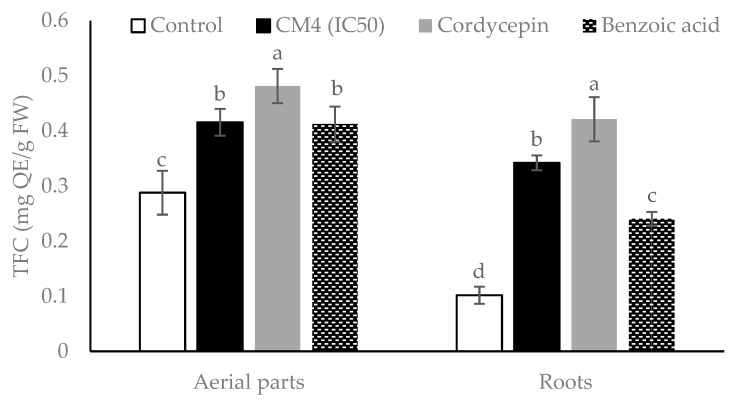
Total flavonoid contents (TFC) of radish among control, CM4 fraction, synthetic cordycepin and BA at 0.04 mg/mL. Columns with different superscript letters (^a,b,c,d^) in the aerial parts and roots indicated significant differences at *p* < 0.05 by Fisher’s test.

Figure 6 shows the changes in the total flavonoid contents (TFC) of the aerial parts and roots of radish seedlings by treatments with CM4, synthetic cordycepin, and BA. The TFC values in both aerial parts and roots are significantly promoted as compared to the controls. The TFC in the roots is increased in greater levels compared to the aerial parts. In every treatment, cordycepin produces the highest TFC, as compared to either CM4 or BA (Figure 6).

#### 2.4.3. Proline Content

**Figure 7:**
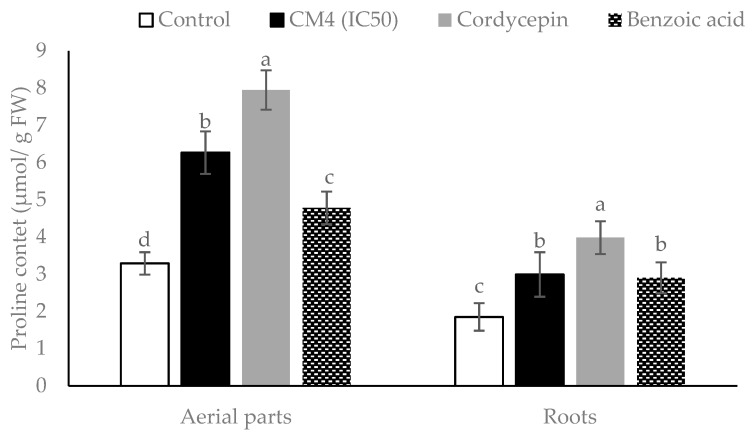
Proline contents in root and aerial parts of radish treated by CM4 fraction, synthetic cordycepin and BA at 0.04 mg/mL. Columns with different superscript letters (^a,b,c,d^) were significantly different at *p* < 0.05 by Fisher’s test.

The responses of proline accumulation in radish due to CM4, BA, and synthetic cordycepin are shown in detail (Figure 7). All treatments significantly stimulate the proline contents in both the aerial parts and roots, as compared with the controls. Treatment by synthetic cordycepin exhibits remarkably higher proline quantities than either CM4 or BA. In addition, the proline content in aerial parts is higher than in roots for all treatments (Figure 7).

### 2.5. Compound Identification by GC-MS

The phytochemical composition of the CM4 fraction (crystal mixture) was determined by GC-MS (Supplementary Materials Figure S1). Among the identified compounds, cordycepin appeared as the dominant component with 55.38% of peak area (*m*/*z*: 251.2), while 1,6-anhydro-beta-d-glucopyranose and pentadecanal were detected with 0.54% and 19.79%, respectively. By GC quantification, the yield of cordycepin in CM4 fraction was 0.226 g (Table 4).

### 2.6. Cordycepin Detection and Quantification by High-Performance Liquid Chromatography (HPLC) and Liquid Chromatography-Electrospray Ionization-Mass Spectrometry (LC-ESI-MS) Analyses

**Figure 8:**
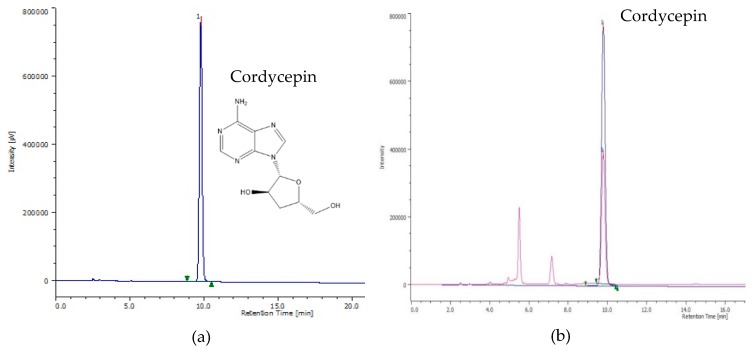
(**a**) HPLC chromatograms of synthetic cordycepin as standard, (**b**) HPLC chromatograms of cordycepin in the CM4 fraction compared with the standard (0.5 mg/mL).

**Figure 9:**
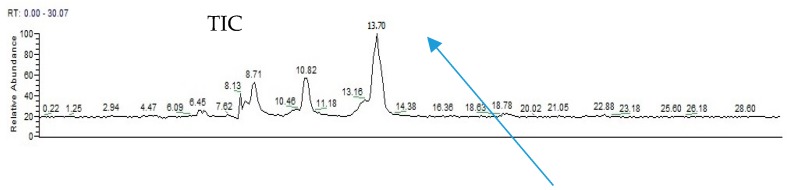

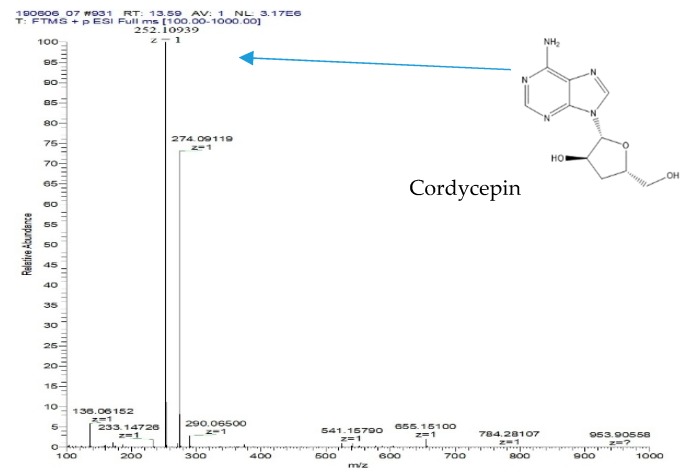
Total ion chromatogram and mass of cordycepin detected in the CM4 fraction.

The HPLC chromatograms of standard synthetic cordycepin and CM4 are presented in Figure 8a,b. Cordycepin was detected at 9.78 min in the HPLC profile of both standard synthetic cordycepin and CM4. The LC-ESI-MS results from Figure 9 confirmed the presence of cordycepin in the CM4 fraction (13.7 min; [M + Na]^+^ *m*/*z*: 274.1; [M + H]^+^ *m*/*z*: 252.1). The use of a positive Fourier transform mass spectrometry (FTMS) mode and certain mass range scans resulted in a total ion chromatogram (TIC) of the fraction, which illustrated a major peak. The retention time and fragmentation patterns from the peak of CM4 were detected as cordycepin, which coincided with that of the standard synthetic cordycepin (Figure S3).

### 2.7. Comparison of Cordycepin Yields in Different Extractions

Table 5 shows a comparison of the cordycepin yields obtained by different extraction methods. Methanol extraction (A) provided the maximum amount of cordycepin (6.166 mg/g DW) as compared to other extracting methods, while the use of a temperature of 70 °C combined with ultrasonic for 30 min provided greater amounts of cordycepin than the extraction using a temperature of 100 °C in 30 min (Table 5). Overall, the use of methanol was more effective than water extraction of cordycepin.

## 3. Discussion

The present study evaluated plant-derived alternatives for globally used agrochemicals, including herbicides such as glyphosate, which has recently been debated and removed from the market in some countries [26]. The focus is on cordycepin, and details were presented on a variety of isolation methods and herbicide characteristics (Figure 1, Figure 2, Figure 3, Figure 4, Figure 5, Figure 6, Figure 7, Figure 8 and Figure 9, Table 1, Table 2, Table 3, Table 4 and Table 5). This study thereby expands previous studies and experience in the area of botanical science including herbicide properties of plants [1,9,26]. Consensus exists among agricultural scientists that chemical herbicides should help provide the world population with enough crops that are free of toxic residues and well tolerated by consumers without causing any health problems. Under these premises, allelopathy can become a promising tool for the sustainable development of agricultural production due to the inhibition of weed growth associated with the prevention of weed resistance to toxic chemicals [27,28]. Released from allelopathic plants, allelochemicals such as phenolics, momilactones, alkaloids, carbohydrates, purines, nucleosides, and amino acids are important sources for natural herbicide development [25,28,29]. Thus, natural products from herbal plants may help decrease the use of synthetic herbicides for weed management, reduce pollution, and provide safer agricultural products of high quality [30].

Experiments of the current study revealed that EtOAc extract of *C. militaris* had the maximum inhibitory effects on the germination, shoot height and root length of radish, as compared to hexane, chloroform, and aqueous residues (Table 1). This suggests that EtOAc extract of fruiting bodies of *C. militaris* might contain principal allelochemicals. In fact, extraction with various suitable solvents can also provide high yields of potent allelochemicals [29]. Among fractions separated by column chromatography eluted by chloroform and methanol (10:0 to 0:10 *v*/*v*), CM4 was the most active fraction to inhibit the germination and growth of radish (Table 2). The analyses of GC-MS and LC-ESI-MS confirmed that cordycepin was the major compound in CM4 (55.38%), followed by pentadecanal (19.8%) (Table 4). Immersion of methanol for two weeks provided more cordycepin than immersion in hot water (70–100 °C) (Table 5), and it was found that repeat boiling of the *C. militaris* fruiting bodies apparently provided greater yields of cordycepin than a single boil. Commonly, cordycepin is isolated from a liquid medium of *C. militaris*. In the present study, we isolated cordycepin from the fruiting body of *C. militaris.* Compared with the previous study that isolated cordycepin from the fruiting body of *C. militaris* [31] by high-speed countercurrent chromatography, our method is more simple and provides a higher yield.

Several factors, such as the type of solvents, temperature, sonication, and extraction time affect the yield of bioactive compounds from plant tissues [32]. In this study, solvent polarity and extraction time may affect the yield of cordycepin in fruiting body of *C. militaris*. Additional solvents combined with different extracting techniques should be tested to receive maximum yields of cordycepin from *C. militaris.*

Allelochemicals inhibit plants directly by affecting their morphology, physiology, and biochemistry [4]. In this study, BA, synthetic cordycepin, and the CM4 fraction containing cordycepin significantly reduced the amount of chlorophyll (a, b, total chlorophylls) and carotenoid of radish as compared with control (Figure 2). Kaya et al. [33] reported that BA reduced pigment contents and decreased the photosynthesis rate. In this study, synthetic cordycepin decreased 2.0- to 9.5-fold the pigment contents of radish compared to BA (Figure 2). These findings suggest that cordycepin possesses stronger phytotoxic activity than BA. Similar to other allelochemicals, both BA and cordycepin may affect porphyrin, a precursor for chlorophyll biosynthesis [34]. Reduction of chlorophylls under allelochemical stress may be caused by impaired chlorophyll biosynthesis, the stimulation of pigment degradation, or both [35].

There are many physiological and biochemical indicators that can be employed to understand how allelochemicals inhibit the receiver plant. Jaballah et al. [36] mentioned that electrolyte leakage, lipid peroxidation, pigment, proline, total phenolic and flavonoid contents are common indicators in response to allelochemical stress. Electrolyte leakage (EL) is one of the strongest indicators of membrane damage in plants effected by allelochemical stress [19]. Increasing membrane permeability could be due to peroxidation of polyunsaturated fatty acids in the bio-membranes, leading to a variety of products including malondialdehyde (MDA) [36]. In this study, MDA accumulation and electrolyte leakage of radish were increased in both the roots and aerial parts when treated with the CM4 fraction containing cordycepin, as well as BA. Chen et al. [37] reported that BA destructs the cell membrane’s integrity due to the formation of free radicals. In this study, cordycepin increased the leakage percentage and MDA accumulation of radish more than BA (Figure 3 and Figure 4).

Total phenolic and total flavonoid contents as well as the proline accumulation in radish, was significantly increased as compared to the control by BA, CM4, and synthetic cordycepin (Figure 5, Figure 6 and Figure 7). Secondary metabolites from plants, such as total phenolics and flavonoids, are structural components of cell walls and participate in defense mechanisms of plants against abiotic and biotic stressors [38]. Ladhari et al. [3] reported that allelochemicals from aqueous and methanolic extracts increase the accumulation of proline in the roots and leaves of lettuce. Flavonoids are the predominant phenolic compounds with important roles as potential inhibitors of the lipoxygenase enzyme, which converts polyunsaturated fatty acid to oxygen-containing derivatives. They accumulate in organs of plants and may help inhibit the process of lipid peroxidation in plants under stress [38]. Similarly, the aerial parts of radish accumulated more proline than the roots. These results are in line with previous research, which reported that the aqueous extract of corn leaves augmented the proline content in wheat leaves [39]. In addition, BA increased the proline content in leaves of tomato seedlings [19] and wheat seedlings [40]. Similarly, in this study synthetic cordycepin induced proline accumulation more than BA in both the roots and aerial parts (Figure 7). Proline synthesis is regulated by various types of stress to allow for the accumulation of proline, a common solute compatible with protective properties [41]. Therefore, increasing the proline level in *R. sativus* may be due to the phytotoxic effects of cordycepin, as well as BA (Figure 7).

Phytotoxic capacity and physiological and biochemical responses of *R. sativus* and some indicator plants to glyphosate have been reported. In a study [42] treating radish plants at 2 mM doses for four days exposure, glyphosate stimulated the shoot height over the control by 20%, but when the dose of glyphosate was increased, this herbicide had a strong inhibitory effect. Compared to our results, synthetic cordycepin inhibited 50% of the shoot height of radish at 0.188 mM dose. Furthermore, in the greenhouse experiment, glyphosate needed more than 600 g/ha dose to decrease chlorophyll a and b of radish from 81.6% to 86.28%, compared with control [43]. In other indicator plants and at a 10 mM dose, glyphosate had a negligible effect on proline accumulation in maize, but EL and MDA increased notably [44]. Similarly, 0.5% (*v*/*v*) glyphosate concentration showed ineffective inhibition of cogongrass [45]. Therefore, cordycepin is more phytotoxic and has a greater effect on the physiological and biochemical processes of receiver plants. With respect to the mode of action, cordycepin functions as an allelochemical compound by inhibiting the germination and growth of radish, reducing pigment synthesis (chlorophylls and carotenoids), stimulating electrolyte leakage, lipid peroxidation, and proline and total phenolic and flavonoid accumulation compared to BA (Table 2; Figure 3, Figure 4, Figure 5, Figure 6 and Figure 7). However, evaluating additional activities of cordycepin, including its functional groups -OH, -NH_2_, and the presence of N on the C4, 7, and 9 of its chemical structure (Figure 8) is required, and respective studies are in progress.

*Cordyceps militaris* is a highly valued edible fungus. It is in use as a dietary supplement in the US and elsewhere, belongs to the large group of herbal TCM (traditional Chinese medicine), and has likely been used safely for centuries in Eastern countries, particularly in China and South East Asia, to treat various diseases [46]. Cordycepin has been well characterized in animal experiments regarding an in vivo subacute toxicity test [47] and Ames test [47], and it is characterized also by rapid decomposition in humans through adenosine deaminase, as evidenced by a short half-life of about 1 min [47]. This likely reduces the possible risk of toxicity and tumor initiation. The chemical structure of cordycepin is very similar to adenosine, with exception of a missing hydroxyl group on carbon number 3 [47]. Therefore, cordycepin can easily be synthetized from adenosine, a natural chemical present in all human cells [48]. The isolation of cordycepin has been described in the literature using various methods [11,16,17,47,48]. The present study improved the isolation procedure, provided a rapid isolation method, and conducted bioassay-guided isolation and identification of plant growth inhibitors from *C. militaris* (Figure 1 and Figure 8; Table 2), resulting in a detailed description of the inhibitory activity of this fungus with its active cordycepin (Table 4). Due to its high water solubility, cordycepin may be problematic for its potential use as a herbicide if it contaminates the ground water. Therefore, respective studies are needed to clarify this issue; it may also be solved by synthesis of a less water-soluble derivate that is still rapidly decomposable in the soil. The overall question remains whether cordycepin can outperform currently used herbicides, which are presently under worldwide dispute despite an impressive list of study results contained in a technical fact sheet on glyphosate [26], but further investigations on cordycepin are also required to exclude any health risk of humans and to carefully evaluate the benefit to risk ratio of cordycepin use. If by error entering the human body, cordycepin will be decomposed within a minute. 

This study shows that *Cordyceps militaris,* with cordycepin as its active component, effectively inhibits plant growth and may be a promising natural source to develop plant-based herbicides. Presently, it is unclear whether cordycepin can outperform glyphosate. Further examinations on the effects of cordycepin on the growth of different and principal weeds in agricultural production, such as *Echinochloa crus-galli* and *Bidens pilosa* are needed. Cordycepin shows at 0.04 mg/mL greater inhibitory efficacy as compared with BA and is thereby basically a promising phytochemical. To clarify the mode of action of this compound, different concentrations of cordycepin as well as its synthesized derivatives, should be examined to detail the physiological and biochemical responses of indicator plants as well as agricultural weeds. The correlation between the phytotoxicity of cordycepin on radish with the corresponding levels of proline, chlorophylls and carotenoids, electrolyte leakage, lipid peroxidation, and total phenolics and flavonoids should be addressed. This would help clarify under what conditions cordycepin could safely and effectively be used as a herbicide.

## 4. Materials and Methods

### 4.1. Reagents

Cordycepin, proline, Folin-Ciocalteu’s phenol, ninhydrin, glacial acetic acid, thiobarbituric acid (TBA), tricholoroacetic acid (TCA), toluene and sulfosalicylic acid were bought from Sigma-Aldrich Japan K.K., Tokyo, Japan. Ethyl acetate, methanol, and methanol plus were provided from Junsei Chemical Co., Ltd., Tokyo, Japan. Hexane, chloroform, acetone, sodium carbonate and aluminum (III) chloride hexahydrate were obtained by Kanto Chemical Co. Inc., Tokyo, Japan.

### 4.2. C. militaris Materials

The fruiting-bodies of *C. militaris* were provided by Truc Anh Company, Bac Lieu city, Vietnam. They were harvested and dried by freeze-drying machine at 15 °C (Mactech MSL1000, Mactech, Hanoi, Vietnam). The dried and sterilized samples were packaged in a sealed container and deposited at 4 °C for further analysis.

### 4.3. Preparation of C. militaris Extracts

*Cordyceps militaris* fruiting-bodies were immersed in water at room temperature for 12 h and dried at 50 °C for 2 days before being pulverized to a fine powder using a grinding machine. The powder (1.0 kg) was soaked in 15 L methanol for two weeks at ambient temperature. The dried crude extract (126.14 g) was mixed with distilled water (500 mL) and then successively fractionated with hexane (C_6_H_14_), chloroform (CHCl_3_) and EtOAc. This fractionation resulted in 10.24 g of C_6_H_14_ (10.25%), 19.25 g of CHCl_3_ (19.28%), 50.21 g of EtOAc (50.27%), and 20.17 g of aqueous residue (20.20%) extracts, respectively. Extract with the highest allelopathic activity was used for further isolation by using column chromatography (Figure 1).

### 4.4. Fractionation of Ethyl Acetate Extract

The EtOAc extract (16.28 g) possessed the highest allelopathic activity on a preliminary test was separated in a normal phase of column chromatography (600 mm height × 40 mm diameter) filled with silica gel (size Ǻ 60, 200–400 mesh particle size, Sigma-Aldrich, Tokyo, Japan). This process yielded eight fractions by the following eluents: CM1 in CHCl_3_, CM2 in CHCl_3_:MeOH (9.5:0.5), CM3 in CHCl_3_:MeOH (9:1), CM4 in CHCl_3_:MeOH (8.5:1.5), CM5 in CHCl_3_:MeOH (7.5:2.5), CM6 in CHCl_3_:MeOH (7:3), CM7 in CHCl_3_:MeOH (6:4), CM8 in CHCl_3_:MeOH (5:5) - MeOH.

### 4.5. Germination and Growth Bioassays

The allelopathic assays were evaluated using the protocol described by Andriana et al. [24]. The sample solutions (300 µL) were pipetted in a plate with 12 wells (35 mm height × 22.1 mm diameter). After methanol was evaporated within 5 h at room temperature, a total of 10 healthy seeds of radish (*Raphanus sativus*) were placed in each well and then added 300 µL of distilled water. The plate was put in a growth chamber. The photoperiod of growth chamber was day/night with a 28/25 °C cycle. After five days, germinated seeds, shoot height and root length were evaluated. The percentages of germination, shoot (hypocotyl) and root (radicle) over the control were expressed as the inhibition percentage (%). The inhibitions on germination and growth of radish were described by the IC_50_ value, which was the amount to suppress 50% of either the germination or growth of radish, therefore a lower IC_50_ indicated a higher allelopathic activity.

### 4.6. Physiological Responses

#### 4.6.1. Chlorophyll and Carotenoid Contents

Chlorophyll (a, b, and total chlorophylls) and carotenoid concentrations of radish seedlings were measured following the protocol reported previously [3]. Briefly, 100 mg fresh weight (FW) of leaves was put in a micro-tube, ground, and added 1.5 mL of acetone (80%). The mixture was centrifuged at 15,000 rpm and supernatant absorbance was recorded at 663, 645 nm and 440 nm by a microplate reader. The applied dose of cordycepin, BA, as well as the CM4 fraction was 0.04 mg/mL (40 ppm). This concentration was used because it was close to the IC_50_ value of the most inhibitory fraction CM4 (Table 2). The contents of all pigments were expressed as µg/g FW with according the formulas:

Chlorophyll a (µg/g) = 12.7 × A_663_ – 2.69 × A_645_ (1)

Chlorophyll b (µg/g) = 22.9 × A_645_ – 4.68 × A_663_ (2)

Total chlorophylls (µg/g) = 20.2 × A_645_ + 8.02 × A_663_ (3)

Total carotenoid (µg/g) = (4.7 × A_440_ – (1.38 × A_663_ + 5.48 × A_645_) (4)

#### 4.6.2. Electrolyte Leakage

The electrolyte leakage (EL) of radish seedlings was evaluated based on the protocol described previously [36]. An amount of 100 mg freshly aerial parts or roots was put in the tube with 15 mL of distilled water. Then, the tubes were stored at room temperature for 24 h, 48 h and the initial electrical conductivity (EC1) was measured by an electrical conductivity meter (EC Meter CM-14P, TOA Electronics Co., Ltd., Nagoya, Japan). The test tube was then autoclaved for 20 min at 121 °C to completely release all electrolytes and quickly reduced to 25 °C. The applied dose of cordycepin, BA, as well as the CM4 fraction was 0.04 mg/mL (40 ppm). The second electrical conductivity (EC2) was recorded. The EL percentage was measured following the equation:

(5) $$\%\;\mathrm{EL}=\left(\frac{{\mathrm{EC}}_{1}}{{\mathrm{EC}}_{2}}\right)\times 100$$

#### 4.6.3. Lipid Peroxidation

Lipid peroxidation was expressed as malondialdehyde (MDA) using thiobarbituric acid (TBA) following the previous method [49]. Fresh samples of roots or aerial parts from 5^th^ day (100 mg) were homogenized in an aliquot of 1.5 mL 0.1% trichloroacetic acid (TCA) and centrifuged at 15,000 rpm for 20 min at 4 °C. A volume of 250 µL of the supernatant was transferred to a test tube, and added 750 µL thiobarbituric acid (TBA, 0.5%) in TCA (20%). The tube was heated for 10 min at 90 °C in a dry bath incubator (MS-100 Thermo Shaker Incubator, On Wing Tat Co. Ltd., Kowloon, Hong Kong). The mixture was cooled down in an ice bath for 5 min. After centrifugation at 10,000 rpm for 5 min at 4 °C, the absorbance of the supernatant was read at 532 and 600 nm by a microplate reader. The MDA quantity was measured using an extinction coefficient (ε = 155 m/M/cm). The applied dose of cordycepin, BA as well as the CM4 fraction was 0.04 mg/mL (40 ppm). The results were displayed as nmol MDA/g FW by using the following formula:

MDA (mM/L) = (A_532_ − A_600_) / ε (6)

### 4.7. Biochemical Responses

#### 4.7.1. Quantification of Total Phenolic Contents

The total phenolic contents (TPC) were measured according to the protocol reported by Quan et al. [50]. Briefly, 100 mg of fresh samples (aerial parts or roots) were extracted with 1.5 mL of MeOH in 24 h and centrifuged at 15,000 rpm for 15 min at 4 °C. Then, the supernatant was used for the total phenolic assay. Briefly, 20 µL of the sample was homogenized 100 µL of the Folin-Ciocalteu’s reagent (10%) and 80 µL sodium carbonate (7.5%), respectively. The absorbance was recorded at 765 nm after 30 min of incubation at ambient temperature. The TPC was presented as mg gallic acid equivalent per g of fresh weight (mg GAE/g FW).

#### 4.7.2. Quantification of Total Flavonoid Contents

The flavonoid contents were determined according to a previous method [51]. In brief, an aliquot of 100 µL of the extract was homogenized with 100 µL aluminum (III) chloride hexahydrate (2% *w*/*v* in MeOH) in a microplate. The absorbance of mixture was read at 430 nm after 30 min incubation at ambient temperature. The TFC was shown as mg quercetin equivalent per g fresh weight of sample (mg QE/g FW).

#### 4.7.3. Proline Content

Proline determination was done as described by Farooq et al. [52]. Briefly, an aliquot of 10 mg of sample powder (aerial parts or roots) was mixed with 1.5 mL of 3% sulfosalicylic acid and centrifuged at 15,000 rpm for 10 min. Then, an aliquot of 250 µL of the supernatant was homogenized with 250 µL of glacial acetic acid and 250 µL ninhydrin reagent (2.5 g ninhydrin in 60 mL glacial acetic and 40 mL 6 M H_3_PO_4_) in a test tube. It was placed in water bath at 100 °C for 1 h and quickly cooled down to stop the reaction in an ice bath. After addition of 500 µL toluene, the upper phase was measured at 520 nm against toluene blank. L-proline (2.5–50 µg/mL) was used as a standard and proline content was expressed as µmol/g FW.

### 4.8. Identification of Phytochemical Constituents by GC-MS and LC-ESI-MS

Phytochemical constituents of the most bioactive fraction were detected by GC-MS and ESI-MS analyses. The GC-MS system was equipped with a DB-5MS column (0.25 mm × 30 m internal diameter, 0.25 µm in thickness) (Agilent Technologies, J & W Scientific Products, Folsom, CA, USA). An aliquot of 1 μL was used for the initial injection. The system used helium as the carrier gas with a split ratio of 5:1. The GC oven temperature started at 50 °C without hold time, final temperature boosted to 300 °C at 10 °C/min for 20 min. The temperature of the injector port and detector were arranged at 300 °C and 320 °C, respectively. The mass was scanned from 29 to 800 amu. The control of system and peak detection process were run based on the JEOL’s GC-MS Mass Center System (JEOL Ltd., Tokyo, Japan) (version 2.65a) [29].

The chemical components in the CM4 fraction were identified by LC-ESI-MS methods. The analysis was performed on a positive/negative ion mode by the system (Thermo Fisher Scientific TM, LTQ XLTM, Ion Trap Mass Spectrometer, Tokyo, Japan). The column J-Pak Symphonia C18 (5 μm, 250 mm × 4.6 mm internal diameter) and mobile phase with 10% acetonitrile in 90% water were used in the LC phase. The volume of fraction injection was 5 μL, and the operation time was 30 min with a flow rate of 0.5 mL/min. System conditions were a sheath gas flow rate (60 arb), ion spray voltage (4.5 kV) and aux gas flow rate (20 arb). The measurements were conducted in a positive mode. MS analyses were conducted using a positive (*m*/*z* 100–1000) Fourier transform mass spectrometry (FTMS) with 60,000 resolutions and negative (*m*/*z* 115–1000) ion trap mass spectrometer (ITMS). Peak processing was conducted using Thermo Xcalibur Qual Browser software (Thermo scientific^TM^, Tokyo, Japan) equipped with NIST MS Library [53]. The presence of cordycepin was determined by comparing their total ion chromatograms (TEC) and mass spectra with those of standard cordycepin.

### 4.9. Quantification of Cordycepin by HPLC

Cordycepin in the CM4 fraction was analyzed according to a method described previously [54]. It was recorded at 260 nm according to the system (LC-Net II/ADC, UV-4075 Plus and PU-4180 Plus, Jasco, Tokyo, Japan). A J-Pak Symphonia C18 column was used with a 250 mm length, 5 μm thickness and 4.6 mm internal diameter. The mobile phase was water (A) 90%: acetonitrile (B) 10%. A gradient elution was used by 0.8 mL/min of flow-rate and 5 μL of sample injection volume. Quantification of cordycepin was conducted by evaluating the peak area based on a standard curve (5, 10, 50, 100 and 250 µg/mL). Cordycepin was identified and quantified by comparing the retention time and peak area of corresponding standard cordycepin.

### 4.10. Cordycepin Content in Different Extractions

To obtain maximum yields of cordycepin, different extraction methods were conducted. Methanol and water were used due to the polarity of these solvents, as cordycepin is a polar compound. Different times, temperatures, and ultrasonic conditions were also employed to obtain optimal yield of cordycepin from fruiting body of *C. militaris* (Table 6).

### 4.11. Statistical Analysis

The statistical analysis was run by one-way ANOVA using the Minitab 16.2.3 software (Minitab Inc., Philadelphia, PA, USA). The values of controls, treatments and standards were expressed as means ± standard deviations (SD). The significant differences between means were examined at *p* <0.05 by using Fisher’s test.

## 5. Conclusions

This study focused on cordycepin successfully isolated from *Cordyceps militaris* and on the discovery of its strong inhibitory potency, which was 3.3- to 3.6-fold greater than glyphosate on the germination and growth of *R. sativus*, as assessed by a variety of laboratory bioassays. Cordycepin, a nucleoside analog (3′-deoxyadenosine) compound and a member of adenosines, caused phytotoxicity as an allelochemical compound by decreasing photosynthetic pigments and increasing electrolyte leakage, lipid peroxidation, proline, total phenolic, and total flavonoid contents. Cordycepin can easily and rapidly be purified using chloroform: methanol (8.5:1.5) as eluent from the ethyl acetate extract of *C. militaris*. The discovery that cordycepin is a novel and potent plant growth inhibitor should encourage the development of plant-based herbicides for environmentally-friendly agricultural production. Further studies are needed to compare the mode of action of cordycepin’s synthesized derivatives compared with glyphosate against the physiological and biochemical responses of indicator plants as well as weeds.

## Figures and Tables

**Table 1:** Inhibition of different extracts of *C. militaris* on germination and growth of radish.

Extracting Solvents	IC_50_ (mg/mL)		
	Germination	Root	Shoot
Hexane	1.388 ± 0.170 ^a^	1.022 ± 0.132 ^a^	0.794 ± 0.043 ^a^
Chloroform	1.250 ± 0.126 ^a^	0.795 ± 0.033 ^b^	0.641 ± 0.436 ^b^
EtOAc	0.235 ± 0.030 ^c^	0.127 ± 0.008 ^d^	0.096 ± 0.006 ^d^
Aqueous residue	0.849 ± 0.082 ^b^	0.410 ± 0.042 ^c^	0.556 ± 0.029 ^c^

Data were presented as means ± standard deviations (SD). Values with different superscript letters (^a,b,c,d^) in a column indicated a significant difference at *p* < 0.05 according to Fisher’s test.

**Table 2:** Effects of different fractions of *C. militaris* on germination and growth of radish.

Fractions	IC_50_ (mg/mL)		
	Germination	Root	Shoot
CM1	2.865 ± 0.247 ^ab^	2.137 ± 0.100 ^a^	1.291 ± 0.183 ^b^
CM2	2.438 ± 0.108 ^bc^	0.561 ± 0.213 ^b^	1.780 ± 0.350 ^a^
CM3	2.090 ± 0.300 ^c^	0.467 ± 0.156 ^b^	1.682 ± 0.204 ^a^
CM4	0.078 ± 0.013 ^e^	0.053 ± 0.004 ^c^	0.052 ± 0.015 ^e^
CM5	1.402 ± 0.121 ^d^	0.382 ± 0.030 ^b^	0.611 ± 0.019 ^cd^
CM6	1.442 ± 0.126 ^d^	0.368 ± 0.205 ^b^	0.862 ± 0.113 ^c^
CM7	2.982 ± 0.449 ^a^	0.466 ± 0.175 ^b^	0.521 ± 0.092 ^d^
CM8	2.463 ± 0.449 ^bc^	0.580 ± 0.076 ^b^	0.523 ± 0.165 ^d^

Data presented means ± standard deviations (SD). Values with different superscript letters (^a,b,c,d,e^) in a column were significantly different according to Fisher’s test (*p* < 0.05).

**Table 3:** Effects of CM4 fraction, BA, cordycepin and glyphosate on the germination and growth of radish.

Treatments	IC_50_ (mg/mL)			
	Germination	Root	Shoot	Reference
CM4	0.078 ± 0.013 ^b^	0.053 ± 0.004 ^b^	0.052 ± 0.015 ^b^	present study
Benzoic acid	0.357 ± 0.052 ^a^	0.183 ± 0.017 ^a^	0.180 ± 0.004 ^a^	present study
Cordycepin	0.061 ± 0.001 ^b^	0.041 ± 0.003 ^b^	0.047 ± 0.004 ^b^	present study
Glyphosate *	0.226	0.137	0.161	[25]

Data presented means ± standard deviation (SD). Values with different superscript letters (^a,b^) in a column were significantly different according to Fisher’s test (*p* < 0.05). * Germination and growth bioassays of glyphosate on radish were conducted in a petri dish assay.

**Table 4:** Identification and quantification of CM4 fraction by GC-MS.

Compounds	Rt (min)	PA (%)	Chemical Formula	MW (g/mol)	Chemical Class	Quantity (g)
1,6-Anhydro-beta-d-glucopyranose	11.76	0.54	C_6_H_10_O_5_	162,14	Anhydrohexose	—
Pentadecanal	14.52	19.79	C_15_H_30_O	226.40	Fatty aldehyde	—
Cordycepin	21.98	55.38	C_10_H_13_N_5_O_3_	251,24	Nucleosides	0.226

Rt = retention time, PA = peak area, MW = molecular weight, —: measurement was not conducted.

**Table 5:** Comparison of cordycepin content in different extraction methods of *C. militaris* fruiting body.

Code	Fungus Part	Extraction Methods				Cordycepin (mg/g DW)
		Solvents	Extraction Times	Temperatures	Ultrasonic	
A	Fruiting body	Methanol	2 weeks	Ambient conditions	—	6.166 ± 0.021^a^
B	Fruiting body	Water	30 min	100 °C	—	3.548 ± 0.012^c^
C	Fruiting body	Water	30 min	70 °C	40 KHz, 30 min	4.248 ± 0.027^b^

—: not employed; DW: dry weight.

**Table 6:** Comparison of cordycepin content in different extractions.

Code	Fungus Part	Extraction Methods			
		Solvents	Extraction Time	Temperature	Ultrasonic
**A**	Fruiting body	Methanol	2 weeks	Ambient condition	—
**B**	Fruiting body	Water	30 min	100 °C	—
**C**	Fruiting body	Water	30 min	70 °C	40 Hz, 30 min

—: not employed.

## Supplementary Materials

Figure S1. GCMS chromatograms of CM4 fraction from EtOAc extract of *Cordyceps militaris*, Figure S2. Mass spectra of the isolated cordycepin from *Cordyceps militaris*, Figure S3. Total ion chromatogram and mass spectra of standard cordycepin.
