# Supplementary material for: Cordycepin Isolated from Cordyceps militaris: Its Newly Discovered Herbicidal Property and Potential Plant-Based Novel Alternative to Glyphosate
Source: Molecules. 2019 Aug 9;24(16):2901. doi: 10.3390/molecules24162901 (PMC6720702; doi:10.3390/molecules24162901)
Supplement: Supplementary file 1 [file molecules-24-02901-s001.pdf]

molecules

MDPI

Article

Cordycepin Isolated from *Cordyceps militaris*: Its newly discovered herbicide property and potential plant-based novel alternative to Glyphosate

Tran Ngoc Quy [1,2], Tran Dang Xuan [1,*], Yusuf Andriana [1], Hoang-Dung Tran [3], Tran Dang Khanh [4,5] and Rolf Teschke [6]

SUPPLEMENTARY DATA

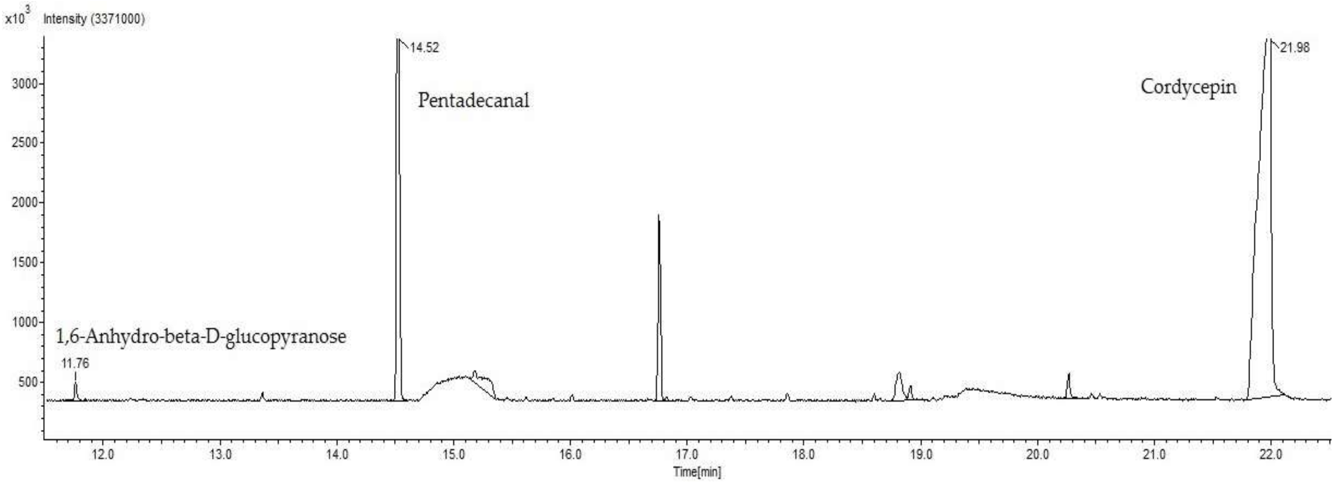

Figure S1. GCMS chromatograms of CM4 fraction from EtOAc extract of *Cordyceps militaris*

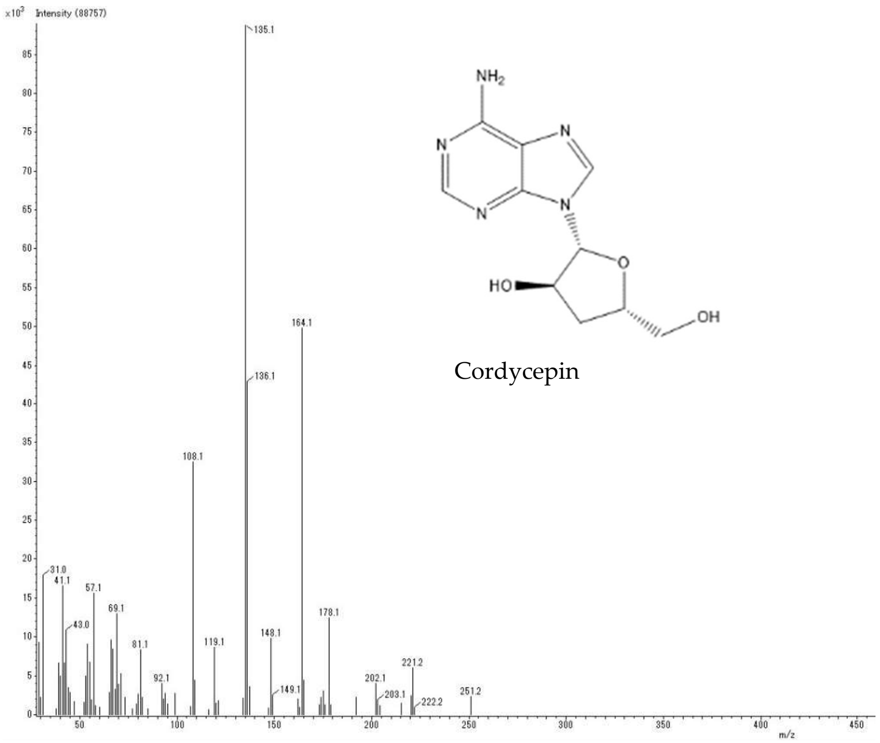

Figure S2. Mass spectra of the isolated cordycepin from *Cordyceps militaris* identified by GC-MS

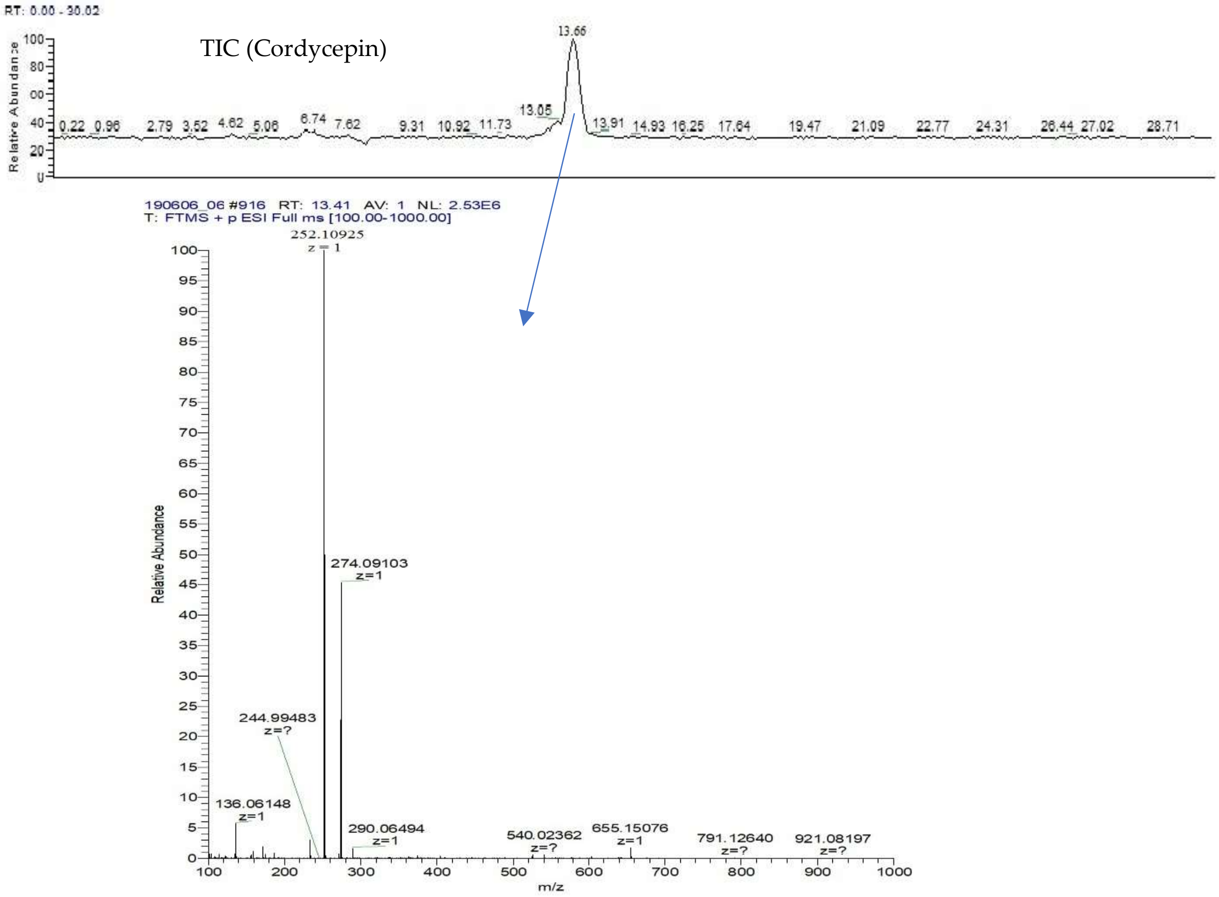

Figure S3. Total ion chromatogram and mass spectra of cordycepin identified by LC-ESI-MS
